# Case Report: Next-Generation Sequencing in Diagnosis of Atypical Aspiration Pneumonia

**DOI:** 10.3389/fpubh.2021.771154

**Published:** 2022-01-04

**Authors:** Quan Zhang, Wei Xu, Fei Peng, Si Lei, Yanjun Zhong, Jun Yi, Shangjie Wu

**Affiliations:** ^1^Pulmonary and Critical Care Medicine, The Second Xiangya Hospital, Central South University, Changsha, China; ^2^Department of Neurology, The First People's Hospital of Chenzhou, Chenzhou, China; ^3^Critical Care Medicine, The Second Xiangya Hospital, Central South University, Changsha, China; ^4^Department of Gastroenterology, Xiangya Hospital of Central South University, Changsha, China

**Keywords:** COVID-19, aspiration pneumonia, next generation sequencing—NGS, case report, differential diagnosis

## Abstract

Since the pandemic of Corona Virus Disease 2019 (COVID-19), especially in the centers most affected, the symptoms such as fever, cough, myalgia or fatigue, and radioactive signs typically related to COVID-19 like ground-glass opacity (GGO) often distract the attention of physicians from other diseases. Aspiration pneumonia and COVID-19 share similarities in some aspects. There may be risk of misdiagnosis in the case of considering radiological patterns of pneumonia. Early diagnosis and treatment often greatly improve prognosis. We herein reported a case of 40-year-old patient who underwent chest CT scan with the discovery of ground-glass opacity, intralobular reticular opacity and interlobular septal thickening, consolidation, and air bronchogram sign, which were mainly located in the middle and upper lobes of the right lung. It was considered to be infection related pneumonia based on the negative reverse transcription-PCR (RT-PCR) result of severe acute respiratory syndrome coronavirus 2 (SARS-CoV-2). The next-generation sequencing (NGS) of bronchoalveolar lavage fluid (BALF) was performed and detected nucleic acid sequences of *Klebsiella sp*. Consequently, the patient accepted sensitive intravenous antibiotics therapy for 13 days and had a remarkable clinical and radiological improvement. His case was followed up through imaging procedures. Because of possible radiologic and clinical similarities between aspiration and COVID-19 pneumonia, COVID-19 can be of some value in proposing a differential diagnosis of aspiration pneumonia. Clinicians could suggest a correct diagnosis by careful examination of the CT images together with attention to the clinical history and judicious utilization of NGS, especially.

## Introduction

Since the outbreak of Corona Virus Disease 2019 (COVID-19), millions of families worldwide have been invaded, and the development of economy, education, health, and other industries in many countries has been affected in varying degrees. In absence of specific therapeutic vaccines or drugs, early detection and isolation becomes essential against the novel coronavirus. However, in that current emergency, with limitations of sample collection or transportation and kit performance, the total positive rate of reverse transcription-PCR (RT-PCR) for throat swab samples was reported to be about 30 to 60% at initial presentation (1). Therefore, radiologists revealed that chest CT can provide benefit for diagnosis of COVID-19. Despite its high sensitivity, CT findings are unspecific and may overlap with other diseases (2). However, the symptoms such as fever, cough, myalgia or fatigue, and radioactive signs typically related to COVID-19, like ground-glass opacity (GGO), often distract the attention of physicians from other diseases.

Aspiration pneumonia is a common disease caused by aspiration or inhalation of irritant substances. It is estimated to account for 5–15% of people diagnosed with community-acquired pneumonia (CAP) and is expected to occupy a larger proportion in the aging society over the next coming decades (3, 4). In general, its CT findings are simply characterized by gravity-dependent lung opacity. However, there are some similarities between aspiration pneumonia and COVID-19 pneumonia. Bilateral subpleural patches of GGO, multifocal patchy consolidation, especially in a peripheral distribution with bilateral, and multifocal lower lung involvement have been described as typical for the diagnosis of COVID-19 pneumonia in suspected cases (5, 6). Such presentations are also fairly common in aspiration pneumonia. These changes, which had been present in up to 74% of the patients, make the discrimination more difficult (7). Therefore, how to realize an effective differentiation of the two diseases and make an initial decision for appropriate allocation of the patients to the COVID or non-COVID wards or intensive care units (ICUs) was a challenge we encountered, especially in the current situation of the persistent outbreak of COVID-19 pandemic. In this context, an early, timely, and accurate diagnosis is utmost important for the treatment of potentially catastrophic and life-threatening infection along with for the prognosis of patients with infection. Here, we report one case of aspiration pneumonia in the atypical site, which was confirmed to be infected by *Klebsiella pneumoniae* (*Klebsiella sp*.). Clinical characteristics and findings of high-resolution thoracic CT of the patient are also described.

## Case Presentation

A 40-year-old Chinese man with smoking (30 cigarettes per day) and drinking (300 ml 52% Chinese rice wine per day) habit for over 10 years, and with 2-month exposure history in COVID-19 epidemic area of Wuhan hospital presented to our Emergency Department on June 28, 2020. The chief complaints of this patient were fever and cough for two days, along with ten bouts of abdominal pain and diarrhea. In addition, the patient experienced shortness of breath since the morning of the day of consultation and still had a fever. For the monitoring of vital signs at admission, the patient presented with a blood pressure of 78/46 mmHg, pulse rate of 122 beats/min, respiratory rate of 35 breaths/min, body temperature of 39°C, and oxygen saturation of 79% on room air. Further physical examination revealed dry skin but cold limbs, several spider nevi in the neck, and moist crackles in the right lung on auscultation. For his epidemic history, the emergency physician immediately prescribed throat swab test for COVID-19. The results of routine laboratory tests showed white blood cell count of 3.73 × 10^9^/L (94% neutrophils), platelet count of 32 × 10^9^/L, procalcitonin of 66.5 ng/ml, creatinine of 182.7 μmol/L, alanine aminotransferase (ALT) of 175 U/L, aspartate aminotransferase (AST) of 416 U/L, lactic acid of 5.6 mmol/L, and Na+ of 132 mmol/L with no rest abnormalities in electrolytes.

**Figure 1:**
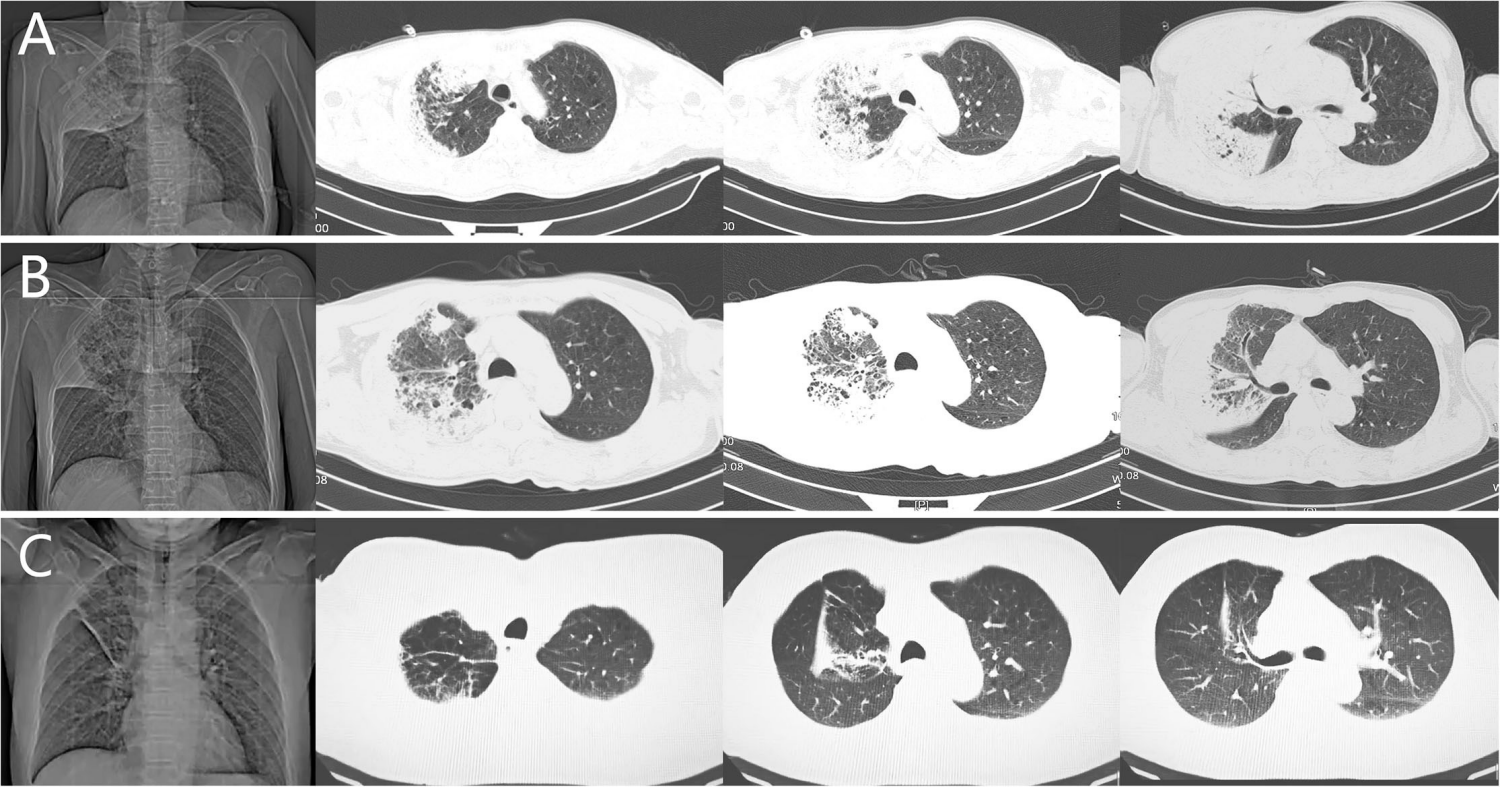
Serial roentgenograms and chest CT images of the patient we reported. **(A)** Transverse thin-section CT of right upper lobe showed ground-glass opacity (GGO), intralobular reticular opacity and interlobular septal thickening. Consolidation and the air bronchogram signs were also presented in the right middle lobe of the patient (on admission). **(B)** Chest CT of the right upper lobe revealed consolidation with cavitation and GGO, with the air bronchogram signs partially absorbed (on day 12 after admission). **(C)** Follow-up CT images on July 4, 2021. The consolidation with cavitation of the right upper lobe were nearly absorbed, but fiber strands were left in the right upper and middle lobe.

The patient was provided with chest CT, with the observation of large pieces of high-density shadows particularly dominant in the middle and upper lobes of right lung (Figure 1A). The patient appeared with a pale face, was restless, and showing signs of severe respiratory distress and was thus provided with endotracheal intubation for ventilation. Subsequently, when the RT-PCR result for severe acute respiratory syndrome coronavirus 2 (SARS-CoV-2) returned negative the second morning, the patient was admitted to ICU for further monitoring and therapy. At first, the vomiting history of the patient was not collected due to sedation. Intravenous norepinephrine and hydrocortisone (100 mg q12 h) were given due to the lack of fluid infusion space to reverse shock. Under the view of bronchofiberscopy, the patient was found to have moderate stenosis of right middle and upper lung bronchus and suctioned with copious yellow bloody secretions from the airways. We chose meropenem (1 g q12 h) and moxifloxacin (0.4 g qd) as the antibiotics for empirical antibacterial treatment, for it was difficult to judge whether the pathogen was gram-positive or gram-negative bacteria. At the same time, the blood sample and bronchoalveolar lavage fluid (BALF) were collected to perform metagenomic next-generation sequencing (NGS) and bacterial culture on the first day. The bioinformatics pipeline included (1) standard sample quality filtering, (2) elimination of replicate reads, (3) removal of low-complexity reads matching human genome sequence, and (4) classification of reads by simultaneously aligning to Pathogens metagenomics Database, which was downloaded from latest RefSeq:NCBI reference consisting of 4,945 whole genome sequence of viral taxa, 6,350 bacterial genomes or scaffolds, 1,064 fungi related to human infection, and 234 parasites associated with human diseases.

**Figure 2:**
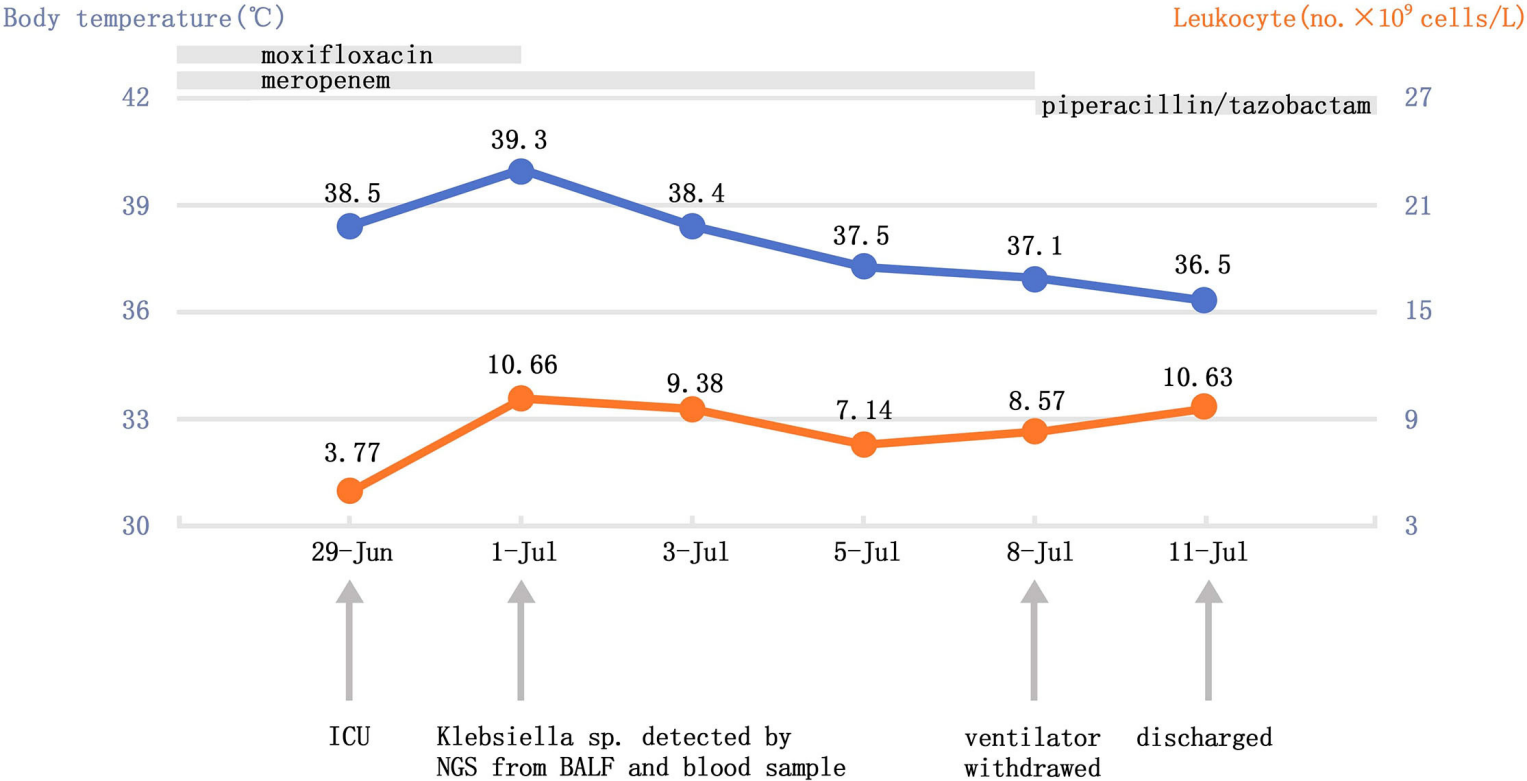
Timeline with relevant data from the case in our hospital; curves of body temperature and leukocyte counts. The arrows below indicate major events. Blue line shows body temperature values. Orange line shows leukocyte counts. ICU, critical care unit; NGS, next-generation sequencing, BALF, bronchoalveolar lavage fluid.

After 48 h on admission, both the NGS of BALF and whole blood sample revealed *Klebsiella sp*., which was consistent with the bacterial culture result of BALF 3 days later. The other micro-organisms detected in the NGS were not regarded as responsible for the invasive pulmonary infection (Table 1). Taking into account the clinical manifestations, rapid organ damage, the NGS test, and CT results, the patient was diagnosed with aspiration pneumonia infected by *Klebsiella sp*. Accordingly, we suspended moxifloxacin. After 8 days of monotherapy with meropenem (1 g q8 h), the patient received an adjusted treatment by piperacillin/tazobactam (4.5 g q8 h) after the body temperature of the patient returned to normal, improvement of laboratory findings, and withdrawal of the ventilator. His mental state had markedly improved and the patient was successfully transferred from the ICU to the respiratory ward on July 8, 2020. Finally, he was smoothly discharged 3 days later when CT scans were in remission (Figure 1B) even though he was left with slight paroxysmal dry cough (Figure 2). Long-term follow-up is currently ongoing to monitor changes in the lung lesion of the patient (Figure 1C) and the patient still has symptoms of intermittent cough 1 year later.

**Table 1:** The results of next-generation sequencing (NGS) in the case.

**SPECIES**	**BALF**		**Blood**	
**Name**	Sequence number^*^	**Relative abundance**	Sequence number^*^	**Relative abundance**
*Klebsiella pneumoniae*	23623	7.71%	897	4.77%
*Klebsiella quasipneumoniae*	693	0.23%	26	0.14%
*Klebsiella aerogenes*	671	0.22%	18	0.10%
*Klebsiella variicola*	670	0.22%	24	0.13%
*Klebsiella oxytoca*	164	0.05%		
*Escherichia coli*	603	0.20%	30	0.16%
*Salmonella enterica*	164	0.11%		
*Citrobacter freundii*	84	0.03%		
*Cedecea neteri*	71	0.02%		

* *The sequence number of the strict comparison of the microorganism detected at the level of species*.

## Discussion and Conclusions

In the setting of COVID-19 pandemic, considering that the patient was living in the epidemic area for 2 months, emergency physicians carried out RT-PCR of SARS-CoV-2 at the very beginning without any hesitation. While waiting for PCR results, the chest CT of the patient showed some radiological signs related to COVID-19, such as GGO, consolidation, and intralobular reticular opacity. Of course, they deployed a single isolation room for the patient. However, during this period, the patient deteriorated and had to receive ventilation. Notably, the treatment and monitoring were relatively limited in the Emergency Department. When the patient was admitted to ICU and received more direct examines along with the NGS test, it was only then that we further proposed a possible diagnosis of aspiration pneumonia despite how no definite history of aspiration was provided and no specific inhalant was found under fiberoptic bronchoscope. Coincidentally, after repeated questioning later on, the patient finally admitted to having several bouts of drunken vomiting 3 days before the onset of symptoms.

Aspiration pneumonia mainly occurs in patients with neurological diseases, disturbance of consciousness, esophageal diseases, trauma, etc., which may cause the irritant substances to enter the respiratory tract and induce secondary inflammation, resulting in an impaired natural defense mechanism. Aforementioned conditions contribute to a large number of admissions to emergency departments. There are currently multiple difficulties for clinical management of aspiration pneumonia, such as the lack of accurate diagnostic standards. The challenge in specifically diagnosing aspiration pneumonia is that for many patients in the community who are at risk for macroaspiration, there is difficulty to get the history of events that occurred in the days leading up to the onset of fever, cough, and infiltrating shadows displayed by chest radiograph. To some extent, chest CT is used to evaluate pulmonary lesions, which is of great importance in the diagnosis of aspiration disorders. Studies have shown that CT manifestations of aspiration pneumonia mainly include atelectasis, centrilobular nodules, bronchiolectasis, consolidation, “tree in bud,” and GGO (8). In addition, these cases can be classified into the following patterns of pneumonia based on CT: lobar pneumonia, bronchopneumonia, bronchiolitis, or others (e.g., pneumonia with interstitial changes, abscess formation) (7). However, these CT findings have been categorized as “atypical signs” of COVID-19 by Radiological Society of North America (RSNA) (9). In view of the diversity and complexity, the imaging findings of aspiration pneumonia are numerous with the absence of specificity. However, due to the special anatomical position of the right bronchus, it is suggested that the location sites of pulmonary findings are more prevalently distributed in the right lung (10) and in lower lung lobes (7). Posterior segments of the upper lobes and superior segments of the lower lobes are frequently involved when patients aspirate in the recumbent position, whereas the bibasilar segments, particularly, the right middle lobe and lingular segment, are affected in patients in their upright position (11–13). Quite a few cases occur in the right middle and upper lobes at the same time, which, however, is the case of our report.

At present, the pathogenic bacteria of aspiration pneumonia have changed from anaerobic to gram-negative bacteria, and the most common bacteria are, in fact, enteral gram-negative species, including *Escherichia coli, Klebsiella sp*., and *Pseudomonas sp*. (14). According to a retrospective study from Japan, CT findings of GGO was most frequent in patients with concurrent pneumonia than those with Klebsiella pneumonia alone, followed by consolidation and intralobular reticular opacity (15). In previous studies, alcoholism was the most commonly associated condition, followed by a smoking habit, cardiac disease, and malignant disease (15, 16). Our case was nearly the same as reported in a recent study as well (17). Interestingly, our case is consistent with two of these conditions described above. Nevertheless, *Klebsiella sp*. are major pathogens in healthcare-associated infections, and their importance appears to be increasing. It has been reported that patients with Klebsiella sp. chest infection frequently suffer a rapid fatal outcome (18). In terms of CT manifestations of *Klebsiella sp*. pneumonia, the previous report mostly showed necrosis or cavity (19), due to this bacteria being associated with pyogenic lung necrosis and pathological processes that result in frequent cavitation. But recent studies revealed that CT findings in patients with acute *Klebsiella sp*. pneumonia consisted mainly of GGO (100%), consolidation (91.4%), and intralobular reticular opacity (85.9%), which were found in the periphery (96%) of both sides of the lungs (72.2%) and were often associated with pleural effusion (53%) (16). In terms of treatment, antibiotics are mainly administrated according to PK/PD principle, but it is necessary to be alert to carbapenem-resistant *Klebsiella sp*. Furthermore, no randomized controlled trials have so far reported the positive effect of glucocorticoid, and it is not recommended in the routine treatment of aspiration pneumonia (20). Moreover, glucocorticoids have been shown to promote the *in vitro* growth of Streptococcus pneumoniae and certain gram-negative rods (21). However, in the treatment process of this patient, glucocorticoid was applied to prevent shock rather than pneumonia.

Next-generation sequencing (NGS) may serve as a perfect one-step tool to study a broad range of pathogen characteristics and is applicable on identifying multiple known or new pathogens (viruses, bacteria, fungi, or parasites) from cultures or directly from clinical samples (22). In addition, NGS is also helpful in detection of novel resistance genes in bacteria, both in current and in historical strain collections. Novel variants of antimicrobial resistance genes, disease transmission, and virulence can be identified or predicted using NGS and further experiments (23). In this case, the patient was initially suspected with COVID-19 on admission and given isolation. Meanwhile, based on the physical condition of the patient being classified as an emergency, to some extent, the treatment of patients has been delayed. After all, the Gram-negative bacteria, Klebsiella sp., that caused shock were quickly identified through NGS technology detection, and the patient was saved by antibiotics targeted at the pathogenic bacteria.

In summary, it is difficult to differentiate COVID-19 pneumonia from other types of pneumonia. There may be a risk of misdiagnosis based on radiological patterns of pneumonia only. Therefore, it is not generally recommended to adopt routine CT screening for diagnosis or exclusion of COVID-19, especially under the condition of not-well-controlled COVID-19 pandemic. Correctly triaging these patients is necessary to reduce the risk of infection transmission and alleviate the pressure of emergency department within hospital. On the other hand, our case highlights the importance of clinician awareness of early pathogen diagnosis by NGS of infectious diseases, antibiotic treatment shall be adopted in a timely manner to decrease mortality and improve prognosis.

## Data Availability Statement

The raw data supporting the conclusions of this article will be made available by the authors, without undue reservation.

## Ethics Statement

The studies involving human participants were reviewed and approved by the Ethics Committee of Second Xiangya Hospital, Central South University. The patients/participants provided their written informed consent to participate in this study.

## Author Contributions

QZ and FP were involved in the clinical care of the patient. QZ and WX designed the case report, created figures, and drafted the manuscript. YZ and SL reviewed the manuscript. JY and SW contributed to the revision of the manuscript and led the scientific discussion. All authors read and approved the final manuscript.

## Funding

This study was funded by Emergency Project of Prevention and Control for COVID-19 of Central South University (Grant No. 160260005) and COVID-19 Emergency Project of Changsha Science and Technology Bureau (Grant No. kq2001052).

## Conflict of Interest

The authors declare that the research was conducted in the absence of any commercial or financial relationships that could be construed as a potential conflict of interest.

## Publisher's Note

All claims expressed in this article are solely those of the authors and do not necessarily represent those of their affiliated organizations, or those of the publisher, the editors and the reviewers. Any product that may be evaluated in this article, or claim that may be made by its manufacturer, is not guaranteed or endorsed by the publisher.

## Abbreviations

COVID-19: Corona virus disease 2019
ALT: Alanine aminotransferase
AST: aspartate aminotransferase
GGO: Ground-glass opacity
CT: Computed tomography
BALF: Bronchoalveolar lavage fluid
RT-PCR: reverse transcriptase-polymerase chain reaction.
